# Building capacity for cancer surveillance and public health research: The Cancer Task Force Project for Cooperation in the Caribbean and Aging Research

**DOI:** 10.7189/jogh.09.020304

**Published:** 2019-12

**Authors:** Clarisse Joachim, Jacqueline Veronique-Baudin, Vincent Vinh-Hung, Cédric Contaret, Jonathan Macni, Lidvine Godaert, Patrick Escarmant, Karim Farid, Jean-Luc Novella, Moustapha Drame, Guillermo Tortolero-Luna, Priscila Torres Babie, Diego E Zavala, Yaima Galan Alvarez

**Affiliations:** 1 UF1441 Registre des cancers de la Martinique, Pôle de Cancérologie Hématologie Urologie, CHU Martinique, Fort-de-France, Martinique; 2 Pôle de Cancérologie Hématologie Urologie, CHU Martinique, Fort-de-France, Martinique; 3 UF 3163, Délégation de la Recherche et de l’innovation, CHU de Martinique, Martinique; 4 Pôle de Gériatrie, CHU de Martinique, Fort-de-France, Martinique; 5 Pole d'imagerie Médicale Service de Médecine nucléaire, CHU Martinique, Martinique; 6 Faculté de Médecine, EA 3797, Université de Reims Champagne- Ardenne, Reims, France; 7 Département de Médecine Interne et Gériatrie, CHU de Reims, Reims, France; 8 Unité d'aide Méthodologique, Pôle Recherche et Santé publique, CHU de Reims, Reims, France; 9 University of Puerto Rico, San Juan, Puerto Rico; 10 Puerto Rico Central Cancer Registry, Comprehensive Cancer Center, San Juan, Puerto Rico; 11 Instituto Nacional de Oncología y Radiobiología, Habana, Cuba; 12 Registro Nacional de Cáncer de Cuba, Sección Independiente para el Control del Cáncer, Instituto Nacional de Oncología y Radiobiología, Habana, Cuba

A total of 6.6 million cancers occurred worldwide in elderly people aged >65 years old (47.5% of all cancers) [1]. The Caribbean is a set of 30 territories with different languages and social systems over a widely dispersed geographical area largely comprised of islands. A total of 90 801 new cancers were estimated in 2012 in the Caribbean; 46 910 estimated cancers for subjects over 65 years of age (51.6%) [1]. Although several cancer management options are currently available, further studies are needed to evaluate the clinical management of patients and patterns of care of elderly patients. Most clinical trials were performed with under-representation of elderly patients. Research is also needed into cancer inequalities, surveillance of infection-related cancers, the role of environmental factors and specific exposures, or identification of the factors that determine health states (such as the structure of the health care system in each country), in patients suffering from cancer in the Caribbean zone [2].

The context in which health care is delivered, and the functioning of the public hospital service in each country are key points to consider in understanding cooperative processes. External factors, both direct and indirect (political, economical, social and environmental) that affect health care delivery and influence the project are also of prime importance for the project’s success. They also partially constitute the hypotheses that condition the achievement of the objectives laid down. Detailed knowledge of current health care policy and delivery, as well as of forthcoming health care reforms, is necessary for the project to be successfully rolled out nationally and internationally.

Four challenges are shared by individual Caribbean territories in health care: isolation and distance; scarcity of medical resources; size of territories and skills in low volume centers; difficulties to reach the break-even point for mandatory high-technology equipment. Capacity building is a ‘process of individual and institutional development which leads to higher levels of skills and greater ability to perform useful research” [3]. Such cooperative approaches could help to bridge disciplines and break down cancer management differences between Caribbean countries, by evaluating patterns of care of cancer patients.

We present the history of the Cancer Task Force Project for cooperation in the Caribbean and Aging Research. This project underlines the importance of innovative and cooperative projects with countries of the Caribbean involved in sharing knowledge and new research approaches.

## PUBLIC HEALTH COOPERATION IN THE CARIBBEAN: FOCUS ON CANCER MANAGEMENT

Since 2015, Martinique has been contributing to the development of centers of excellence in terms of public health for the OECS, to achieve economies of scale, spreading of knowledge, increased experience, and synergy in research, with a view to improving the quality of care and health care delivery.

A joint approach is a solution to overcome cancer health care challenges within the Caribbean: sharing knowledge and skills, training in oncology; improving decision making through telemedicine; facilitating local treatment and avoiding transfer of patients; sharing equipment. The introduction of multidisciplinary meetings that are accessible from a distance through telemedicine is a major step forward that could be replicated in neighbouring countries, especially for complex cases. Digital sharing of health data are therefore the first stage towards constituting a group of experts, specialists in medical oncology. Our experience from the last three French national strategic plans of cancer could be shared on cancer management guidelines and multicentre observational studies on cancer treatments modalities, such as the utility of consulting medical and paramedical announcers, post-cancer programmes, Multidisciplinary Consultative Meetings for the Caribbean & e-consultation, or Oncogeriatric Units for the elderly.

**Figure:**
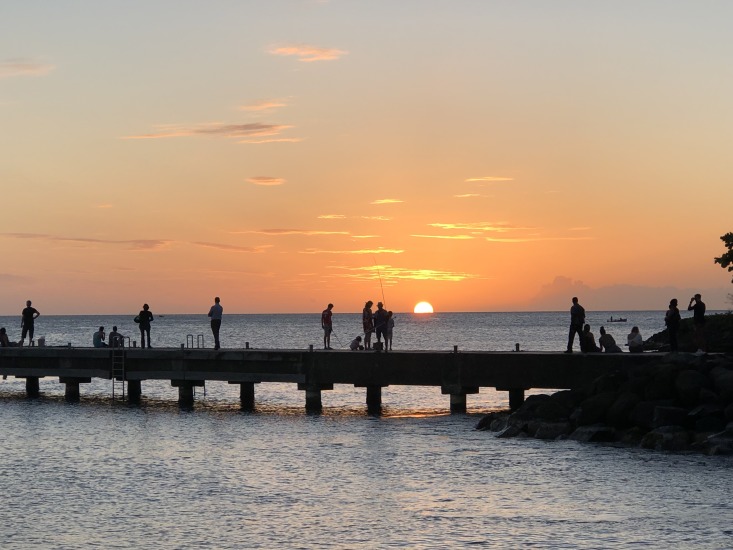
Photo: Building bridges between countries, a key success factor for the understanding of the elderly populations of the Caribbean (from the collection of Clarisse Joachim, used with permission).

In Puerto Rico, a strategic plan for cancer provides a framework and guide for coordinated and integrated efforts to reduce the burden of cancer. Many cancer priorities along the cancer continuum have already been identified and are being addressed in some way. These include which cancers are the most common, which ones cause the greatest mortality, which areas in the Island have the greatest disparities, what are those disparities, and what groups in the population are the most affected. The plan proposes evidence-based or theory-based strategies and also catalyzes existing partnership and potential partners to take action. It serves as a focal point for island-wide efforts in cancer prevention and control.

In Cuba, the national public health system’s cancer prevention and control strategy was introduced in 1987, as the National Cancer Control Program (NCCP) with the aim of lowering cancer incidence and mortality, and increasing survival and quality of life. In 2010, the newly-designed programme and national strategy for cancer control was fully set in the country as a platform to integrate cancer control actions throughout the National Health System [4]. The strategy is organized through four axes namely: 1) education/communication; 2) early detection/primary health care; 3) diagnosis and treatment/ supportive care; and 4) research/evaluation and is deeply integrated into one of the main programmes of the Cuban health system: the Family Doctor and Nurse Programme, and the network of hospital services for diagnosis and specialized treatment. The national network of comprehensive cancer care is organized according to different aspects, such as: levels of complexity in care, specialized services, geographic regions, levels of activity and clinical sites. It also includes a new component not found in the earlier stages of the Cuban Cancer Program, namely the impact of biotechnology.

The plan prioritizes work in four areas nationwide:

1. Seven types of cancer: lung, female breast, prostate, colorectal, uterine cervix, oral cavity and childhood cancers;
2. Development and control of cancer therapies and resources (radiotherapy, clinical and surgical oncology, nuclear medicine, natural and traditional medicine and biotechnology products);
3. Social determinants associated with cancer;
4. Broad participation in cancer control.

In the face of all these health care challenges, data-sharing for the Caribbean zone is subject to international data protection laws. Healthcare data are a prime example of personal data, and are considered as particularly sensitive. For this reason, there are specific dispositions in the law for protecting health care data in order to protect the privacy of citizens. With the globalization of data exchanges and the increasing use of new technologies, the number of data transfers is constantly rising. Transfer of data outside of the country in which the data originated is possible, provided the necessary measures are taken to ensure sufficient, appropriate protection of that data. Such data transfers are strictly regulated by various judicial means. For example, the details of the transfer, and in particular the categories of personal data and the purposes for which they are transferred, are specified in research protocols between countries. For international cooperation projects, public health care establishments may also sign agreements with partners from the public or private domain, in compliance with the international commitments of each of the participating partner countries.

The creation of joint, anonymised databases for research purposes must be in compliance with these regulatory and legislative constraints, in order to perform modelisation and analysis of data in each of the participating partner countries in the project. For the purposes of clinical research, agreements stipulating the specific conditions for exchange of data (how, in what form, by whom, etc) have been developed.

## ROLE OF POPULATION-BASED CANCER REGISTRIES IN SCIENTIFIC COOPERATION

To develop sustainable and impactful research programmes on cancer in this region, the Cancer Task Force Project for Cooperation in the Caribbean and Aging Research started in 2017 with Martinique, Puerto Rico and Cuba. Systematic recording of all cancer cases is essential in order to produce reliable epidemiological data, and is necessary to improve management and reduce mortality. Thanks to site visits already carried out in 2015, cooperative relationships have been established with Puerto Rico, Cuba and countries of the Organisation of Eastern Caribbean States, as well as with French West-Indies cancer registries. Priorities were established through a participatory process by identifying research themes common to participating partner countries, and classification of the projects by priority within the teams; this initiative was promoted during scientific meetings and workshops to explain the role of population-based cancer registries and what activities are ongoing and could be shared with the Caribbean.

The Martinique Cancer Registry (MCR) has been participating in epidemiological surveillance and evaluation of cancer since 1981, through the analysis of incidence and mortality data. Thanks to exhaustive and continuous data collection for all cancer cases, key indicators are calculated to study the trends in different types of cancers across the region of Martinique. Incidence and mortality studies for each type of cancer, as well as prognostic studies are also initiated and performed to describe the spatial and temporal distribution of the different cancer types. The MCR contributes to international epidemiological knowledge through chapters produced by the International Agency for Research on Cancer. The last contribution was in the Cancer Incidence in Five continents Volume XI available at http://ci5.iarc.fr. Data of the registry were also analysed in the Cancer survival study CONCORD III [5]. Several collaborative exchanges were pursued in 2015 with the Puerto Rico Central Cancer Registry (PRCCR) and National Cancer Registry (NCR) of Cuba. These registries have thus demonstrated their capacity to establish public health missions in the framework of cancer surveillance in the Caribbean, given the long experience and the excellent quality of these Registries in the Caribbean zone.

The PRCCR is an agency of the Department of Health established in March 1950 and is responsible for collecting, analyzing, and publishing information on all cancer cases diagnosed and/or treated in Puerto Rico. In October of 1997, the PRCCR initiated its participation in the US National Program of Cancer Registries (NPCR) coordinated by the Centers of Disease Control, of the United States’ Department of Health and Human Services. In 2014, the PRCCR launched its new webpage at: www.rcpr.org. Puerto Rico’s Cancer Registry participation in international studies includes all editions of the Cancer Incidence in Five Continents and International Incidence of Childhood Cancer, Vol II and Vol. III, both published by the International Agency for Research on Cancer (IARC). The PRCCR data was approved and included in the international cancer survival study CONCORD II and CONCORD III. Several public health schools and cancer researchers are the main users of PRCCR data. The PRCCR Research and Analysis Unit work closely with all researchers in projects, presentations, and publications; however, the Registry also produces its own publications including bi-annual cancer incidence reports.

The NCR of Cuba, covers the whole population of the country, was created in 1964 and upgraded in 1986 to include mandatory reporting and improved data management systems. The information system was decentralized in 1994 to each provincial level. Since 2004 the Network of the Cancer Registries has started to work.

The Cancer Registry Network of Cuba meets physically once a year, and holds virtual meetings once a month where topics of general interest are discussed and methodological and administrative guidelines are issued. This Registry is developing software for the handling and processing of data, ie, SISCAN, which should start to be used in 2018. This will lead to an update of the data collection model and the procedures manual. An increase of the quality of the data was observed using an own tool developed to check it. The NCR of Cuba has participated in several cooperative projects with the IARC, REDEPICAN and Latin American countries and has extensive experience in the use of its data for clinical and epidemiological research, for the planning of resources and in the monitoring and evaluation of the activities of the Cancer Control Program. Likewise, the NCR data have been used for the planning of human and material resources.

## FACTORS CONTRIBUTING TO SUCCESSFUL COOPERATION

International cooperation meets local needs in each of the participating countries, and these needs that have to be taken into account in order to ensure that the partnership is appropriate and effective. These needs arise from public health challenges that are common to cancer surveillance and aging. Indeed, the constant and rapid increase in the number of elderly persons, combined with particularly rapid population aging in the Caribbean, represent a major public health issue. Over the coming 20 years, the Caribbean area will see its population age rapidly, with the number of persons aged over 60 set to almost double, from 1.1 million (13%) in 2015 to 2 million in 2035 (22%). The number of persons aged over 70 will increase from 500 000 to one million. In the face of this changing demographic profile, sharing experience with international partners to focus on scientific collaboration, and particularly research projects, will make it possible to pool resources from different sources to meet the health care challenges of tomorrow.

Structuring exchanges and identifying needs in terms of health care training, as well as training of health care professionals in medical cooperation are all important elements for ultra-peripheral regions like ours. Our work towards establishing a map of cooperative processes will help modelize health care data at the level of the Caribbean.

Furthermore, our project identified key players from the target countries through collaborative projects conducted by visio-conference and through on-site visits. Inter-registry meetings held during the 40th IACR Annual Scientific Conference in November 2018 in Arequipa, Peru, were instrumental in cementing the projects and its fundamental objectives. This conference brought together all the partner teams from Cuba and Puerto Rico, with the possibility to hold working meetings on site to develop a research programme and work towards scientific publications.

## CANCER IN THE ELDERLY IN THE CARIBBEAN: THE NEED FOR ACTION IN AGING RESEARCH

Cancer is a disease mainly affecting elderly people, nevertheless few data exist on cancer care of elderly patients in real-life practice in the Caribbean [6-9]. Geriatric oncologists have to evaluate the risk-benefit ratio of cancer treatments, balancing life expectancy while maintaining quality of life in elderly patients. International recommendations indicate that treatment could be adapted to clinical profiles of elderly patients. These patients are more frequently treated with a non-curative approach and may be vulnerable to treatment side effects. Observational cohorts in elderly patients could help to describe the burden of cancer, with subgroup analyses on stage at diagnosis, treatment modalities or comorbidities. However, there is a compelling need for action in the Caribbean, due to the increasing health care inequalities among elderly people. Due to the side effects of treatment, assessment of quality of life has become an important priority in cancer care management in developed countries. The EORTC QoL-Group and the Cancer in the Elderly Task-Force specific to QoL are working on questionnaires in elderly cancer patients; such initiatives could be extended to the Caribbean population for Quality of Life research projects [10]. Accordingly, the European Organization for Research and Treatment of Cancer (EORTC) specific questionnaires on quality of life (EORTC QLQ-C30) and on prostate cancer (Prostate cancer module QLQ-PR25) will be proposed to patients. Given the high incidence of prostate cancer in the Caribbean, this project will enable us to compile, for the first time, high-quality data about quality of life in prostate cancer, based on data from population-based cancer registries.

## CREATION OF A CANCER SURVEILLANCE PLATFORM FOR KNOWLEDGE TRANSFER AND RESEARCH

**Figure 1:**
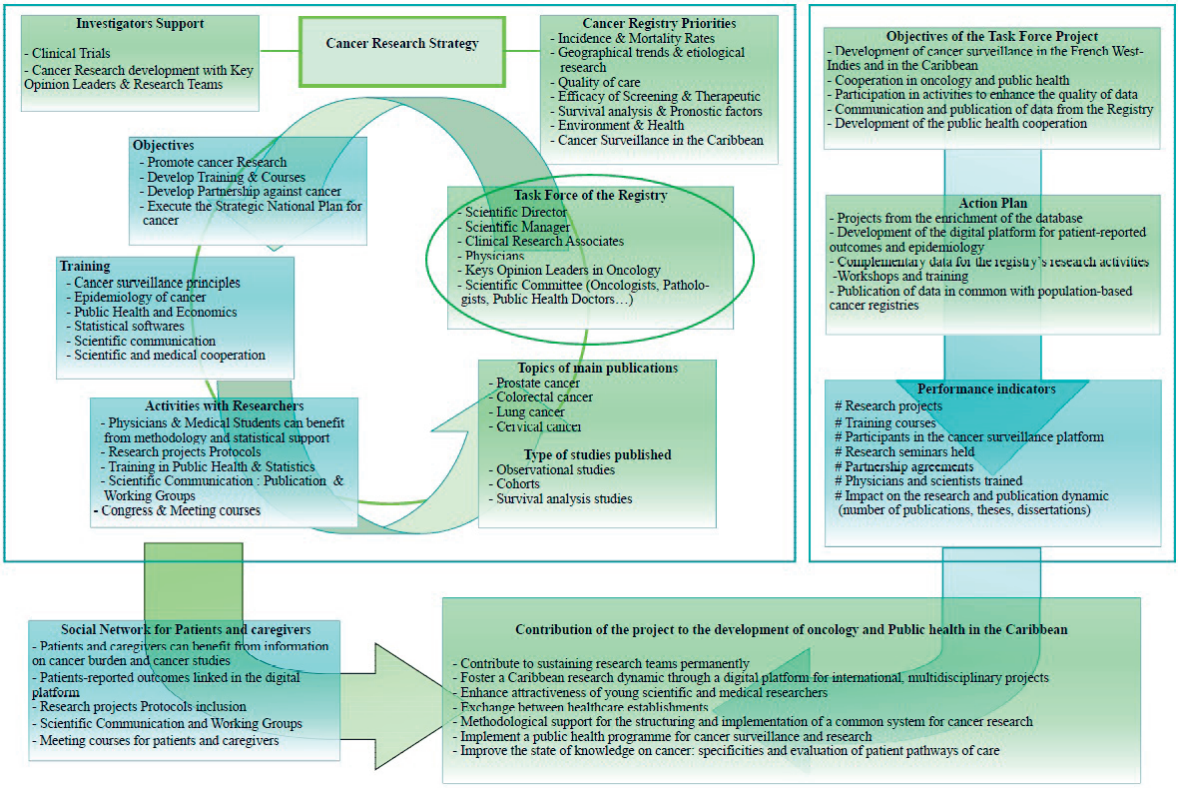
Strategic plan of the Cancer Task Force Project for the Caribbean and Aging Research.

The MCR proposed to provide systematic support for scientific meetings to spread the knowledge and national experience coming out of our registries. A digital information leaflet identifying the strategic plan (**Figure 1**) for cancer surveillance and research projects will be developed in English and Spanish, to be shared with cancer research teams of the Caribbean. This platform could also serve for communication, methodological mentoring, and teaching, and make it possible to promote data from the Caribbean. It could also serve as the interface between the Caribbean on the one hand, and Europe on the other hand. This is an innovative and structuring project that has made it possible to usher in a new phase of active development of research projects on themes that are specific to the Caribbean region.

This project concerns the following priorities for knowledge transfer and research:

Priority 1: Reinforce health care systems while fighting disease

This project will help to reinforce systems for cancer surveillance through the Caribbean zone by making available public health indicators that are common to several countries in the Caribbean, all of whom have a Cancer Registry.

Priority 2: Promote population health

Developing informatics systems for health care professionals, institutions and scientific partners, as well as patients, through the use of a digital exchange platform will contribute to actions designed to promote health in the Caribbean area.

Priority 3: promote expertise, training, research and innovation

Training health care personnel is a major component of this project. Implementation of training in public health, oncology and haematology in the Caribbean zone will meet the demands of health care professionals, and reduce inequalities in access to training and research programmes.

The main objectives are:

1) to promote improved knowledge in public health and epidemiology, and their communication thanks to e-health initiatives, in the framework of an inter-regional and pan-Caribbean strategy for research in public health and cooperation in cancer;

2) To participate in the evaluation of public health measures and patient pathways for patients with cancer in the Caribbean, in collaboration with other structures responsible for coordination of cancer care, quality of care and safety.

## CONCLUSIONS

Population-based cancer registries have a role as an independent and essential expert in the evaluation of public policy. In collaboration with other regional public health structures, they represent a key instrument in the follow-up of regional health care plans, through contributions to the regional prevention scheme, and the regional health care organisation outline. The Cancer Task Force Project for Cooperation in the Caribbean and Aging Research will make it possible to identify populations at risk and help to reduce inequalities among cancer patients. Management patterns in patients with cancer will be analysed, taking into account vulnerable populations, especially elderly patients. We implement a communication strategy targeting the Caribbean and aimed at raising awareness among the states of the region about research achievements of the cancer registries and their researchers working throughout the Caribbean.

This article underlines the importance of creating a consortium of researchers within the Caribbean to develop priority research goals for elderly persons suffering from cancer, based on data from population-based Cancer Registries. The Cancer Registries of Cuba, Puerto Rico and Martinique are among the oldest in the Caribbean, and contain high-quality data that can contribute to international knowledge on the epidemiology of cancer in the Caribbean region. The strategic action plans to fight cancer that are present show the perspectives for cooperation through the implementation of multicentre research projects in the future. The creation of the “Cancer Task Force Project for cooperation in the Caribbean and Aging Research” underscores the importance of innovative approaches to cooperation for datasharing and biotechnology transfer in the domain of health. This federative project for the Caribbean region could subsequently be extended to other countries participating in cancer surveillance.
